# Solid polymer electrolyte-based atomic switches: from materials to mechanisms and applications

**DOI:** 10.1080/14686996.2024.2342772

**Published:** 2024-04-16

**Authors:** Tohru Tsuruoka, Kazuya Terabe

**Affiliations:** Research Center for Materials Nanoarchitectonics (MANA), National Institute for Materials Science (NIMS), Tsukuba, Japan

**Keywords:** Atomic switch, nanoionics, solid polymer electrolyte, resistive switching, moisture absorption, inkjet printing, quantum conductance, neuromorphic computing

## Abstract

As miniaturization of semiconductor memory devices is reaching its physical and technological limits, there is a demand for memory technologies that operate on new principles. Atomic switches are nanoionic devices that show repeatable resistive switching between high-resistance and low-resistance states under bias voltage applications, based on the transport of metal ions and redox reactions in solids. Their essential structure consists of an ion conductor sandwiched between electrochemically active and inert electrodes. This review focuses on the resistive switching mechanism of atomic switches that utilize a solid polymer electrolyte (SPE) as the ion conductor. Owing to the superior properties of polymer materials such as mechanical flexibility, compatibility with various substrates, and low fabrication costs, SPE-based atomic switches are a promising candidate for the next-generation of volatile and nonvolatile memories. Herein, we describe their operating mechanisms and key factors for controlling the device performance with different polymer matrices. In particular, the effects of moisture absorption in the polymer matrix on the resistive switching behavior are addressed in detail. As potential applications, atomic switches with inkjet-printed SPE and quantum conductance behavior are described. SPE-based atomic switches also have great potential in use for neuromorphic devices. The development of these devices will be enhanced using nanoarchitectonics concepts, which integrate functional materials and devices.

## IMPACT STATEMENT

This article reviews a series of works starting with the author’s 2011 paper on solid polymer electrolyte-based atomic switches, and describes the current status and future prospects for this technology.

## GRAPHICAL ABSTRACT

**Figure:**
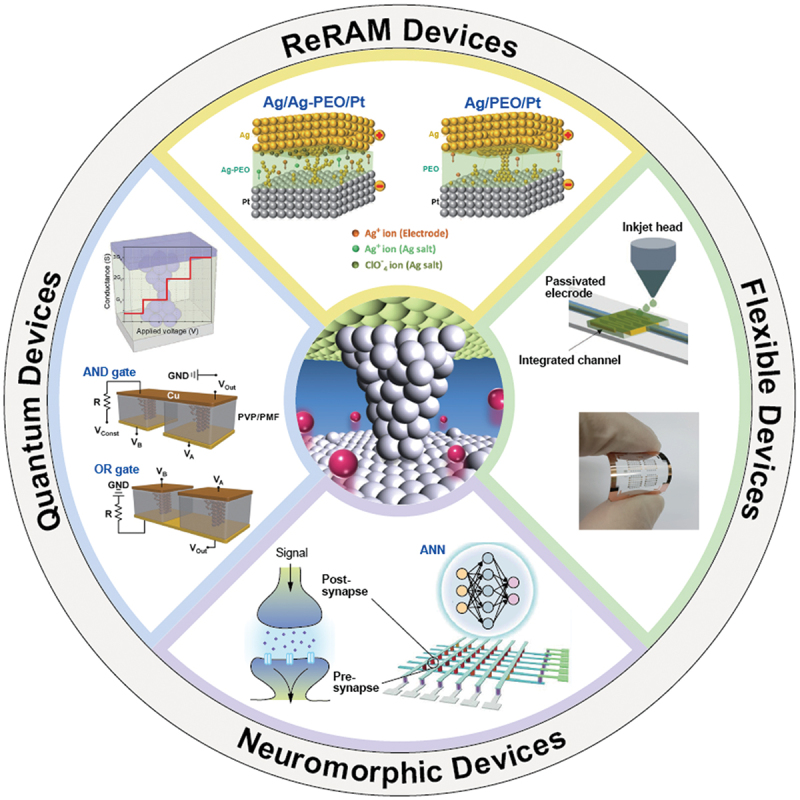

## 1. Introduction

Over the past several decades, information and communication technologies (ICTs) have developed in an evolutionary manner, supported by the continuous downscaling of semiconductor integrated circuits (ICs). As Moor predicted [1], the number of transistors on ICs has doubled approximately every 2 years. However, as the device downscaling approaches the physical, technological, and economical limits of complementary metal-oxide-semiconductor (CMOS) devices [2], new solutions for nonvolatile and volatile storage are being investigated. Such investigations are taking place all over the world, not only on the research level but also on the industry level. Resistive random-access memory (RRAM) is one of the most promising alternatives to current memory technologies such as flash memory and dynamic random-access memory, because of its simple structure, high scalability, and lower power consumption [3].

Of the various RRAMs available, nanoionic devices that utilize local ion transport and redox reactions in solids have attracted much attention as components that can extend or replace current memory technologies. Such devices are categorized as redox-based RAM (ReRAM) [4,5]. About 20 years ago, our group proposed a conceptually novel ReRAM device called an ‘atomic switch’ [6,7]. The first such atomic switches used a nanometer gap between a mixed conductor electrode and an inert metal electrode, where resistive switching between a low-resistance (ON) state and a high-resistance (OFF) state can be controlled by the formation and annihilation of a metal bridge in said gap. Resistive switching based on the same principle was found to be possible for a metal/electrolyte/metal stacked device, in which an electronically insulating but ionically conducting film is sandwiched between an electrochemically active metal electrode (usually Ag or Cu) and an inert metal electrode (e.g. Pt or Au). This type of stacked device is also known as an electrochemical metallization (ECM) cell [8] or a conductive bridge RAM (CBRAM) [9]. To date, various materials have been examined for use as the electrolyte matrix, such as chalcogenides [10,11], metal oxides [12–15], and organic molecules [16]. Selection of the electrolyte material is crucially important if stable and desired functions are to be obtained in atomic switches and ReRAM devices.

In 2009, we theorized that atomic switches could be realized using a solid polymer electrolyte (SPE). SPEs have high ionic conductivity with negligible electronic conductivity, exhibit high mechanical flexibilities and stabilities up to their melting points, and have excellent processability onto a variety of substrates. Many SPE materials have been developed for various applications, including not only batteries [17] but also supercapacitors [18] and smart sensors [19], in which alkali-metal (Li^+^ and Na^+^) salts are incorporated [20]. However, alkali-metal salts are generally highly reactive under ambient conditions, and are therefore not suitable for memory devices. We considered that Ag salts are more stable than alkali-metal salts [21], and accordingly began research on SPE-based atomic switches using Ag salts. In this endeavor, we had the help of Dr Katsuhiko Ariga and Dr Johnathon P. Hill, who are experts in the supramolecular chemistry of various organic materials [22,23]. The study of atomic switches using high ionic conductivity electrolytes was also important in elucidating the operating mechanism of atomic switches using low ionic conductivity electrolytes like metal oxides [24,25]. At around the same time, a research group at NEC Corp. reported resistive switches that utilized another type of SPE for nonvolatile programmable logic circuits [26,27]. In their works, SPE was designed so as to withstand the lithography processes, like resist materials (detailed information about the material was not disclosed). As a result, the ionic conductivity of the SPE used is considered to be low at room temperature, which is different from our material design.

Various RRAM devices have been developed using various materials and operating mechanisms, supported by the progress in nanotechnology related to material science. About 10 years ago, new attempts began to successfully connect these devices to each other in order to realize advanced functionalities in architectures such as perceptual sensory devices, artificial neural networks, and neuromorphic computing [28,29]. This approach can be regarded as being based on the emerging concept of ‘nanoarchitectonics’, which is a post-nanotechnology concept that integrates functional materials and devices using the knowledge and protocols of nanotechnology [30]. For example, utilization of the interactions between nanoionic devices has led to successful demonstration of center-surround antagonism of the retina [31] and neuromorphic systems imitating human functionalities such as decision-making [32]. Decision-making utilizing SPE-based atomic switches was also theoretically predicted [33]. In order to develop such nanoarchitectonic systems, a fundamental understanding of the operating mechanism of atomic switches with various SPE materials is very important.

In this review, we report on the current achievements of the research on SPE-based atomic switches (and ReRAMs) by addressing several major issues, including their operating mechanism and the key factors that determine switching behaviors. First, in Section 2, the operating mechanism of SPE-based atomic switches is described, showing typical resistive switching behaviors and direct observations of the corresponding filament growth processes. In Section 3, the resistive switching behaviors of different SPE matrices are described so as to elaborate on the effect of the crystallinity of the polymer material. In Section 4, based on the results for different SPE materials, we explain the impact of moisture absorption in the polymer matrix on the resistive switching behavior. In Section 5, we present the potential applications for SPE-based atomic switches, flexible switch/memory, and quantum conductance (QC) devices. This section also shows the results of the first-principles transport simulations that account for the origin of the observed quantum conductance, and the neuromorphic applications of SPE-based atomic switches are briefly discussed. Section 6 is comprised of the Summary and Outlook.

## 2. Operating mechanisms of SPE-based atomic switches

**Figure 1.**
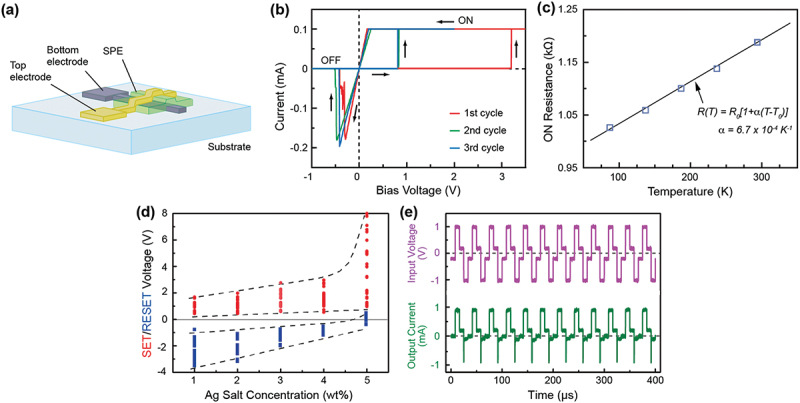
(a) Schematic of the fabricated cross-point SPE-based atomic switch. (b) Typical *I-V* curves of a Ag/Ag-PEO/Pt device with 3 wt% and ~1 µm thick Ag-PEO film formed by drop casting. (c) ON resistance temperature dependence, showing the metallic conductivity. *α* is the temperature coefficient of the resistivity, which is obtained by fitting to the experimental data. (d) SET and RESET voltages plotted as a function of the Ag salt concentration. (e) Write-read-write-read cycle test of a device with 2 wt%. The cycle frequency is 30 kHz and the pulse width for writing and erasing is 8.3 µs. Reproduced with permission from [37] copyright 2011, Wiley.

We selected polyethylene oxide (PEO) for the first polymer matrix, because several groups have reported high ionic conductivities in PEO complexed with Ag salts [34,35]. Magistris et al. reported that a PEO electrolyte that includes Cu salt (Cu(ClO_4_)_2_) is essentially an anionic conductor, with a cationic transport number lower than 0.05, as found in similar systems with Pb, Mg, and Ca salts [36]. We therefore considered that Ag is the most suitable salt for the atomic switch applications detailed herein. The devices were fabricated on glass or SiO_2_-covered Si substrates. The SPE solution was prepared using appropriate amounts of the matrix polymer and Ag salt (AgClO_4_), which were dissolved in water or acetonitrile solvent. The device consisted of a cross-point structure with a junction size ranging from 50 × 50 to 5 × 5 µm^2^, with Ag and Pt as the top and bottom electrodes (TE and BE), respectively, as illustrated in Figure 1(a). Figure 1(b) shows typical *I-V* curves for the first three voltage sweep cycles, measured for an Ag/Ag salt-incorporated PEO (Ag-PEO)/Pt device with an Ag salt concentration of 3 wt% [37]. The Ag-PEO film was formed by drop casting, followed by vacuum drying at 120°C, resulting in a film thickness of ~1 µm. The current compliance *I*_*CC*_ was set at 0.1 mA only for positive bias, in order to avoid irreducible damage to the device due to a large ON current. The device initially exhibited a pristine OFF state of greater than 10 GΩ. As the bias voltage was swept in the positive voltage direction, the current suddenly jumped up to the compliance level at a larger bias voltage, which turns on the device with an ON state of a few kΩ. This first SET process, from OFF to ON states, corresponds to the forming process of a conducting filamentary path between the electrodes. The device kept a low resistance even when the bias voltage was returned to 0 V, which is consistent with nonvolatile switching behavior. Subsequent negative bias application resulted in sharp switching of the device back to the OFF state of ~1 GΩ, which corresponds to the RESET process. An ON/OFF resistance ratio up to 10^5^ was obtained. Figure 1(c) indicates that the ON resistance changes linearly with the ambient temperature, suggesting electronic transport in a metal. This implies that the ON state is attributable to the formation of a metal filament [38].

The switching characteristics depend strongly on the Ag salt concentration. Figure 1(d) shows how the SET and RESET voltages change with Ag salt concentrations of from 1 to 5 wt%. With increased salt concentration, the SET voltage slightly increased up to 4 wt% and then spread out over a wide bias voltage range at 5 wt%. The RESET voltages decreased in magnitude with increased salt concentration, with some appearing in the positive bias voltage at 5 wt%, suggesting a transition to volatile switching behavior. For 6 wt% or higher, no resistive switching was observed in a bias voltage range of ±10 V, indicating that there is an optimum range in the salt concentration for repeatable switching. Figure 1(e) shows a typical result for write-read-write-read cycle tests conducted on a device with an Ag salt concentration of 2 wt%. A high switching speed of less than 1 µs was confirmed, indicating the appropriate capabilities of the SPE-based atomic switch for fast operations.

**Figure 2.**
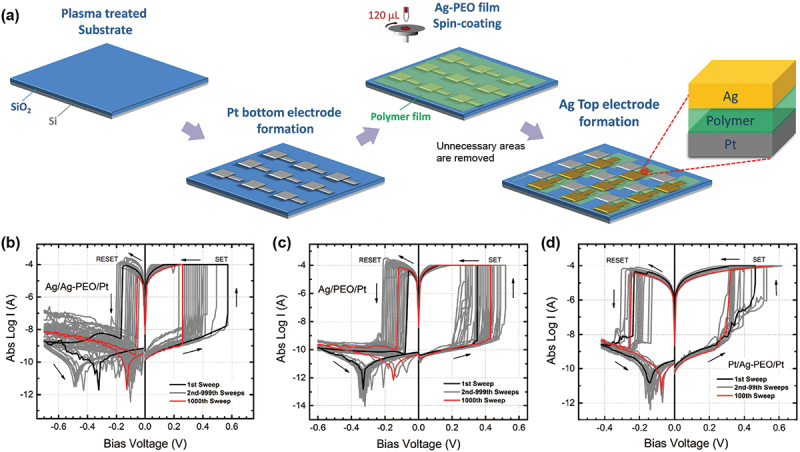
(a) Fabrication scheme of atomic switch devices with spin-coated SPE films. Typical *I-V* curves of (b) Ag/Ag-PEO/Pt, (c) Ag/PEO/Pt, and (d) Pt/Ag-PEO/Pt devices with 3 wt% and 200 nm thick Ag-PEO and PEO films formed by spin coating, obtained for 10^3^ consecutive sweep cycles. Reproduced with permission from [39] copyright 2016, Wiley.

With the drop casting method, the deposited SPE films spread over a wide area, and it is difficult to control the uniformity and thickness less than 500 nm. To overcome this problem, we adapted a spin-coating method to form uniformly thick SPE films. By optimizing the concentration of SPE solution and the spin-coating conditions, we were able to obtain a self-assembled polymer structure, which gives rise to tunable resistive switching characteristics. Figure 2(a) illustrates the fabrication scheme for devices with spin-coated SPE films. After spin coating the SPE films, unnecessary areas were removed so as to isolate each device. Figure 2(b–d) show typical *I-V* curves for Ag/Ag-PEO/Pt, Ag/PEO/Pt, and Pt/Ag-PEO/Pt devices with an Ag salt concentration of 3 wt% and an SPE film thickness of ~200 nm, measured after the forming process [39]. All the devices exhibited stable switching behavior over 10^3^ sweep cycles, as evidenced by the black (first sweep) and red (10^3^th sweep) curves. Although the forming voltage of the Ag/PEO/Pt device was much higher than that of other devices, the SET and RESET voltages were almost the same for all devices. This is taken to mean that, once a filament is formed, the subsequent dissolution and reformation of the filament are likely to take place in a similar way regardless of the material configuration.

It was found that switching characteristics are primarily determined by measurement conditions such as the voltage sweep range and sweep speed. We always took pains to optimize these measurement conditions so as to ensure that the switching behavior remained constant. Under such conditions, the OFF resistance did not decrease significantly during the switching cycle, as is shown in Figure 2(b–d). On the other hand, an important feature of the *I-V* curves in Figure 2 is the current minima observed in negative bias after the RESET operation. These current minima, on a logarithmic scale, correspond to a zero-crossing point on a linear scale, and the bias voltage at this point is associated with electric polarization, which corresponds to an open-circuit (electromotive force, emf) voltage [40,41]. The emf voltage generally originates from the charge accumulation of cations and anions at opposite electrodes, as discussed later in detail. We see that the emf voltage changes dynamically during switching cycles, and its behavior varies from one device to another. It was found that switching stability, as well as the emf voltage, depends strongly on a stop voltage at negative bias. If the bias voltage was swept larger to the negative side, a higher SET voltage was required at the next sweep cycle, giving rise to fluctuations in the switching behavior. This is because the negative stop voltage affects the charge distribution in the polymer matrix after the RESET operation.

To understand the operating mechanism, we performed *in-situ* and *ex-situ* microscopy observations to investigate filament growth processes under voltage applications [39,42]. The high ionic conductivities of SPE enabled us to directly observe filament formation behaviors, even in micrometer-scale devices. For this experiment, planar devices with opposing Ag (or Pt) and Pt (or Ag) electrodes were fabricated on a SiO_2_/Si substrate, using lithography techniques, with gaps between the two electrodes ranging from 0.5 to 10 µm. An Ag-PEO film with an Ag salt concentration of 3 wt% or a pure PEO film, with thicknesses of ~200 nm, were formed on the gap by the spin-coating method. A constant bias voltage (2 V) was applied to the Ag electrode or to one of the Pt electrodes relative to the other Pt electrode, and the current-time (*I-t*) characteristics were measured, and a simultaneous video recording of the morphological change of the devices was made using an optical microscope. A low *I*_*CC*_ (11 nA for the Ag/Ag-PEO/Pt and Ag/PEO/Pt devices and 1 µA for the Pt/Ag-PEO/Pt device) was set to pin the initial growth of the metal filaments. After *I-t* measurements, the morphology of the devices was checked by scanning electron microscopy (SEM).

**Figure 3.**
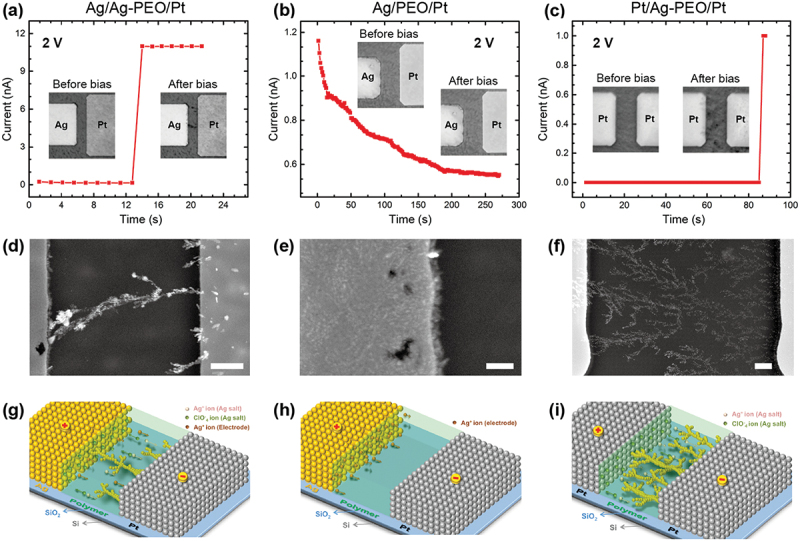
(a–c) *I-t* plots measured under constant voltage biasing of 2 V for Ag/Ag-PEO/Ag, Ag/PEO/Pt, and Pt/Ag-PEO/Pt planar devices, with gaps of 5.6, 8.5, and 8.5 µm, respectively. The insets show i*n-situ* optical microscope images taken before and after voltage biasing. (d–f) SEM image taken after the voltage biasing. The scale bar is 1 µm. (g–i) Schematics of filament growth behavior in the three planar devices. Reproduced with permission from [39] copyright 2016, Wiley.

Figure 3(a) shows an *I‒t* curve measured for the Ag/Ag-PEO/Pt device. The current was slightly decreased for an initial period (~13 s), which arises from the oxidation of Ag at the Ag electrode, before suddenly jumping up to the *I*_*CC*_ according to the appearance of filament structures from the Pt electrode to the Ag electrode, as seen in the insets (optical microscope images). The SEM image obtained after bias application (Figure 3d) shows the formation of a metal filament bridging between the Ag and Pt electrodes, with some Ag precipitations on the Pt electrode. This filament growth behavior can be explained as follows; as a positive bias voltage is applied to the Ag electrode, the edge region of the Ag electrode starts to dissolve, which is evidenced by voids in the SEM image, and the oxidized Ag^+^ ions transport toward the Pt electrode. In addition, the pre-existing Ag^+^ ions in the Ag-PEO matrix also migrate toward the Pt electrode under applied bias. When the Ag^+^ ion concentration in the vicinity of the Pt electrode reaches a certain threshold, precipitation of Ag takes place at many sites on the Pt electrode. Finally, an Ag filament grows from one of the precipitated sites to the Ag electrode, as illustrated schematically in Figure 3(g). The insets show that the filament seems to grow along the phase-segregated structures. However, microscopically, the self-assembled lamellar-like structures of the PEO matrix create interconnected polymer chains, which provide the long-range ion transport pathways [43]. As a result, Ag^+^ ions can migrate preferentially along these pathways, promoting faster and unidirectional filament growth.

Figure 3(b) depicts an *I‒t* curve measured for the Ag/PEO/Pt device. The current decreased gradually with time, corresponding to the Ag oxidation at the Ag electrode, but never reached the *I*_*CC*_ even after several hundred seconds. Similar to what was observed for the Ag/Ag-PEO/Pt device, the SEM image (Figure 3e) shows that the edge region of the Ag electrode was dissolved during bias application, as is evidenced by voids. However, because of the low ionic mobility of the pure PEO, Ag ions can transport only a few hundred nm from the Ag electrode and are precipitated there, as illustrated in Figure 3(h). The Pt/Ag-PEO/Pt device exhibits a completely different filament growth behavior. Figure 3(c) is an *I‒t* curve measured for this device, showing almost the same *I‒t* characteristic as the Ag/Ag-PEO/Pt device. However, the corresponding optical microscope image represents the gradual and random growth of Ag precipitation. The SEM image (Figure 3f) indicates the dendritic growth of Ag precipitation. Similar dendritic growth was also observed for Li metal in Li/PEO-salt/Li cells [44,45]. When a bias voltage is applied to the left-sided Pt electrode, the pre-existing Ag^+^ ions in the Ag-PEO matrix start to migrate toward the opposite Pt electrode. Such randomly distributed Ag^+^ ions in the Ag-PEO matrix make simultaneous random pathways for Ag ion transport toward the grounded electrode. The subsequently precipitated Ag atoms grow along these transport pathways, resulting in the formation of random dendrites from the grounded Pt electrode, as illustrated in Figure 3(i). The SEM image also shows that many portions of the dendrite structures bridge between the two electrodes, indicating multifilament formation.

The above results suggest that filament growth and resistive switching behaviors depend strongly on the device configuration and experimental parameters, such as the salt inclusion in the PEO matrix, the gap distance, and the applied compliance current. These parameters are linked to the kinetic factors that determine the filament growth processes [46]. First, salt inclusion has the most significant impact on the redox reaction rate at the biased electrode, ion mobility in the PEO matrix, and the reduction sites for precipitations. These factors determine the filament formation morphology of unidirectional or dendritic growth processes and the magnitude of the forming voltages. Second, the gap distance determines the electric field strength affecting the filament morphology, and provides the critical limit length for stable switching behavior. Finally, the density and size of the grown filaments are controlled by the *I*_*CC*_ level and determine whether the switching behaviors are volatile or nonvolatile. Different filament formations, resulting from unidirectional or dendritic growth behavior, can be controlled by tuning specified parameters, which in turn improves the stability and performance of SPE-based atomic switches.

A similar SEM observation of the filament formation was reported for an Ag/poly(3,4-ethylene-dioxythiophene):poly(styrenesulfonate) (P2HT:PCBM)/Pt planar device [47]. Conducting filaments were found to consist of partially sulfurized Ag clusters that start to precipitate from the middle region of the gap between the Ag and Pt electrodes, which was attributed to faster diffusion of Ag ions on the surface of the P2HT:PCBM film. Recently, Wang et al. reported the filament growth behavior of an Ag/Li salt included-PEO/Pt planar device by means of *in-situ* optical microscopy and *ex-situ* SEM observations [48]. They found that the precipitation of Ag atoms is related to the crystallinity of the polymer matrix, which affects the filament morphology. The observed variability of resistive switching behavior is ascribed to the variation of the filaments formed in different cycles and devices, i.e. shape, location, and quantity. All the results described here indicate the usefulness of direct observations of planar device structures to elucidate the filament growth behavior in SPE-based ReRAMs.

## 3. Role of the polymer materials on resistive switching

Resistive switching was observed not only in PEO but also in other polymer materials such as polyvinyl alcohol (PVA) and polyvinyl pyrrolidone (PVP). These polymers, known as hydrophilic polymers, have carbon chain backbones with hydroxyl groups attached to methane carbons [49,50]. Such hydroxyl groups participate in the formation of polymer complexes via hydrogen bonding [51]. Due to the strong interaction between water molecules and polymer chains, both PVA and PVP show relatively high conductivity, even below the glass transition temperature *T*_*g*_. A significant amount of research has been done on metal-salt included PVA and PVP complexes, for energy and battery applications, because of their enhanced ionic conductivity by water uptake [52]. We investigated the impact of polymer matrix materials on resistive switching behavior using PEO, PVA, and PVP as the polymer matrices [53]. All the polymer matrices included 3 wt% of Ag salt (AgClO_4_), and their resistive switching characteristics were examined at varied temperatures of between 23°C and 70°C.

**Figure 4.**
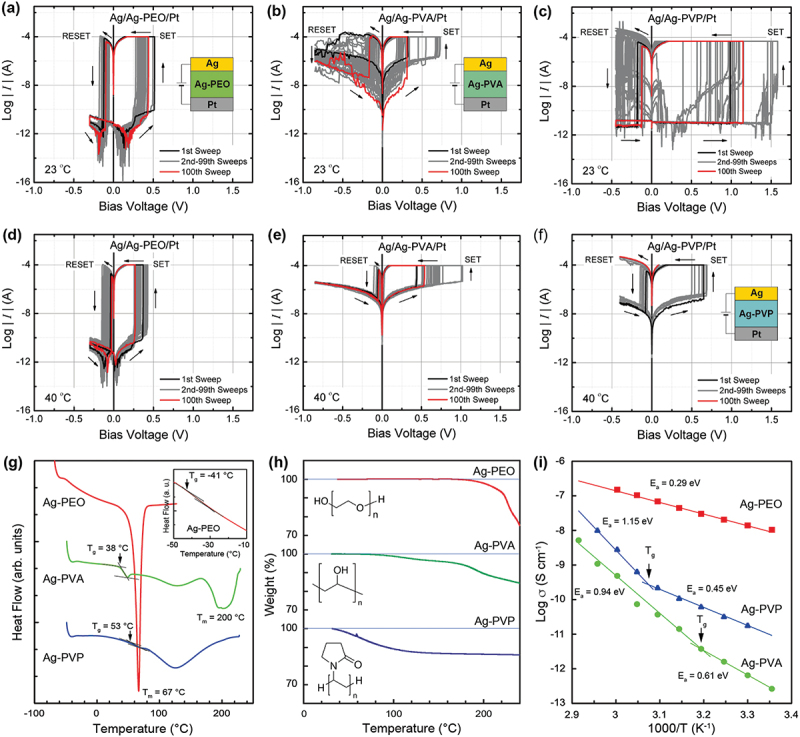
Resistive switching characteristics of (a, d) Ag/Ag-PEO/Pt, (b, e) Ag/Ag-PVA/Pt, and (c, f) Ag/Ag-PVP/Pt devices, measured at 23 and 40 °C. (g) DSC and (h) TGA curves of Ag-PEO, Ag-PVA, and Ag-PVP films. The inset of (g) depicts a magnified view in the temperature range of between −50°C and −10°C, showing the *T*_*g*_ of Ag-PEO. All the curves were obtained at the first scan. The insets of (h) illustrate the molecular structure of three polymer matrices. (i) Temperature dependence of the electrical conductivity of the three SPE films, evaluated from EIS measurements. Reproduced with permission from [53] copyright 2019, Institute of physics.

Figure 4 shows *I-V* curves measured for atomic switches with the three polymer matrices at 23°C and 40°C with *I*_*CC*_ = 50 ~ 100 µA. All the pristine devices exhibited a very high resistance of more than 1 GΩ. After the forming process, the devices exhibited bipolar switching behavior, but each device showed different temperature behaviors. The Ag/Ag-PEO/Pt device showed stable switching at both temperatures for more than 100 sweep cycles (Figure 4(a,d)). In contrast, the Ag/Ag-PVA/Pt and Ag/Ag-PVP/Pt devices exhibited unstable switching at 23°C, with both the SET and RESET voltages fluctuated considerably (Figure 4(b,c)). These devices failed to switch at some cycles, but recovered when the bias voltage was kept sweeping. The stability of such switching behaviors was improved by increasing the temperature to 40°C (Figure 4(e,f)). If the temperature was increased further, the switching behavior of the Ag/Ag-PVA/Pt device became very stable, as reported in [54]. However, the Ag/Ag-PVP/Pt device completely failed to switch at some specific cycle (~25 cycles) and no longer exhibited switching behavior.

To understand the temperature variation observed in different SPE materials, the structural, thermal, and transport properties of the SPEs used were investigated. X-ray diffraction (XRD) results showed that the Ag-PEO film has a crystalline nature, whereas the Ag-PVP film has an amorphous nature. The Ag-PVA film displayed comparative amounts of crystalline and amorphous phases. Figure 4(g) shows typical differential scanning calorimetry (DSC) curves measured for the three SPE films. The *T*_*g*_ was estimated to be −41°C, 38°C, and 53°C for the Ag-PEO, Ag-PVA, and Ag-PVP films, respectively, as indicated by the arrows. The melting temperature *T*_*m*_ was reached at 67°C and 200°C for the Ag-PEO and Ag-PVA films, respectively, whereas no *T*_*m*_ peak was observed for the Ag-PVP film, suggesting that the Ag-PVP film has an amorphous nature with almost no crystalline phase. The *T*_*g*_ observed for the Ag-PVA and Ag-PVP films are much lower than those for pure, dry PVA and PVP films (80 and 150–180°C, respectively). These lowered values are mainly associated with the plasticizing effect of water incorporation into the amorphous region of the PVA and PVP matrices [55]. This is evidenced by an additional broad endotherm appearing on the DSC curves of the Ag-PVA and Ag-PVP films at around 125°C.

Figure 4(h) represents thermal gravimetric analysis (TGA) curves, showing the weight loss of the three SPE films when increasing the ambient temperature up to 240°C. The Ag-PEO film shows almost no weight loss up to 160°C, whereas the weight of the Ag-PVA and Ag-PVP films starts to decrease above 50°C and below 40°C, respectively. This weight reduction is attributed to water evaporating from the amorphous regions of the PVA and PVP matrices. Figure 4(i) presents the Arrhenius plots of the electrical conductivity σ of the three SPE films, which were evaluated from electrochemical impedance spectroscopy (EIS) measurements (a detailed analysis is given later). Of the three SPEs, the Ag-PEO film exhibited the highest conductivity with the lowest activation energy *E*_*a*_. In contrast, the Ag-PVA and Ag-PVP films showed lower conductivities of more than two orders of magnitudes and higher *E*_*a*_. These SPEs have two *E*_*a*_ values, with an inflection point corresponding to the *T*_*g*_ observed in their DSC curves.

From the results obtained, the temperature behavior of resistive switching can be explained as competition between segmental mobility and water evaporation in the polymer matrix. In the crystalline PEO matrix, the *T*_*g*_ is much lower than 0°C, which leads to the polymer matrix exhibiting high ionic conductivity at room temperature, which in turn arises from the highly segmental mobility of the polymer chains. As a consequence, the Ag/Ag-PEO/Pt device displays stable resistive switching characteristics over a wider temperature range up to the *T*_*m*_. In the semi-crystalline PVA matrix, the *T*_*g*_ is found at close to room temperature. This is caused by plasticization due to moisture absorption in the amorphous region, which changes the PVA matrix from a glassy state to a rubbery state and promotes the segmental mobility of the polymer chains. Therefore, the Ag/Ag-PVA/Pt device exhibits resistive switching even at room temperature, below *T*_*g*_. As the temperature rises, the ionic conductivity increases because of enhanced segmental mobility. However, due to water evaporating from the amorphous region in the polymer matrix, the device shows slightly larger SET and RESET voltages at elevated temperatures [54].

In the amorphous PVP matrix, the *T*_*g*_ is also reduced by plasticization of absorbed water, but it is still relatively higher than room temperature. This situation leads to less segmental mobility of the polymer chains, although the ionic conductivity is higher than Ag-PVA. Hence, the Ag/Ag-PVP/Pt device shows unstable resistive switching at room temperature. As the temperature rises, the ionic conductivity increases, in a manner similar to the PVA matrix. However, significant water evaporation takes place in the amorphous region, such that the weight of the polymer matrix is easily reduced by several %, which in turn decreases the free volume for hopping conduction of Ag ions. Because of such significant weight loss, the Ag-PVP device still exhibited unstable resistive switching and failed at a limited number of sweep cycles at higher temperatures. From the perspective of molecular structure, crystalline and semi-crystalline polymers show better performance as matrix materials than amorphous polymers, because amorphous polymers significantly alter their properties by the absorption and evaporation of water from the surroundings.

Various polymer materials have been examined as the SPE matrices, including Ag nanowire-polystyrene composites [56], P3HT:PCBM [57], poly(n-vinylcarbazole) (PVK) [58], chitosan [59], poly(melamine-co-formaldehyde) (PMF) [60], carboxymethyl introduced κ-carrageenan (CM:κ-car) [61], polyethyleneimine (PEI) [62,63], and Li salt-included PEO [48]. Recently, Krishnan and co-workers reported resistive switching behaviors using Ag nanoparticle (AgNP)-included poly(methyl methacrylate) (PMMA) [64] and poly(vinylidene fluoride-co-hexafluoro propylene) (PVdF-HEP) [65]. An Ag/PMMA/Al device exhibited lower SET voltages than an Ag/AgNP-PMMA/Al device, indicating that an inclusion of AgNP promotes the formation of Ag filaments. On the other hand, an Ag/PVdF-HEP/Au device showed that the electron transport is a combined effect of space charge limited, trap-filled charge limited, and ohmic conduction processes, the behavior of which alters with each voltage sweep. The authors attributed this to the high dielectric constant of PVdF-HEP, which induces a unique charge carrier separation at the electrode interfaces. However, in these studies, the effect of moisture absorption was not taken into account. Further investigation is needed to clarify the correlation between the resistive switching characteristics and the structural properties of the SPE matrix.

## 4. Effects of moisture absorption into the polymer matrix

**Figure 5.**
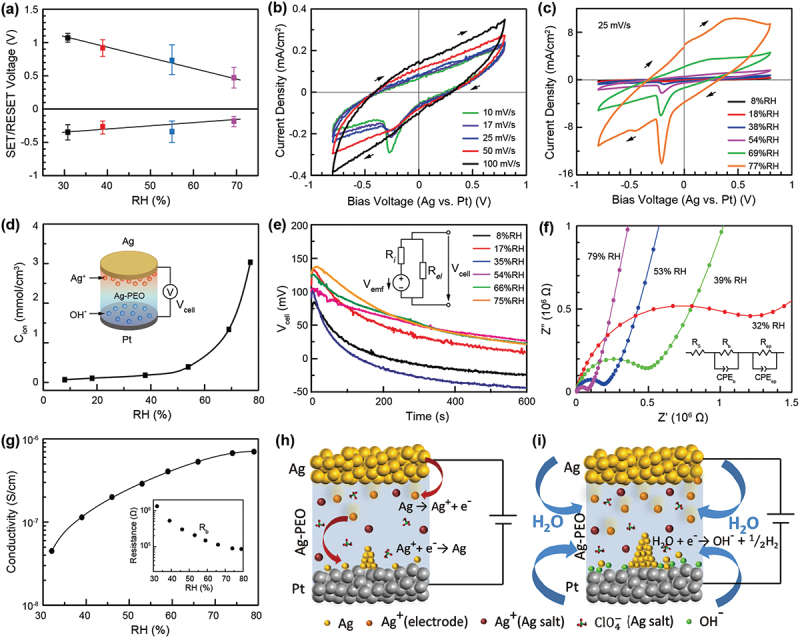
(a) SET and RESET voltages of a Ag/Ag-PEO/Pt device, plotted as a function the RH level. (b) CV curves measured in air at varied sweep rates, from 10 to 100 mV/s. (c) CV curves measured under varied RH levels, from 8 to 77%. (d) *C*_*ion*_ plotted as a function of RH level, evaluated from a single sweep in the positive voltage regime, with a sweep rate of 25 mV/s. The inset illustrates how the *V*_*cell*_ appears relative to the ion concentration gradient generated in the device. (e) Time evolution of *V*_*cell*_ for different RH levels. The inset illustrates the equivalent circuit model under emf generation. (f) Nyquist plots of the impedance response measured for a Ag-PEO film at different RH levels. The inset illustrates the equivalent circuit model describing the observed impedance behavior. (g) Electrical conductivity plotted as a function of RH levels, calculated from the values of R_b_. (h) and (i) Schematics illustrating the effect of moisture absorption on the switching behavior of a Ag/Ag-PEO/Pt device under low and high RH conditions, respectively. Reproduced with permission from [66] copyright 2021, Royal Society of Chemistry.

As described in the previous section, resistive switching behavior significantly alters with the use of various polymer materials with different crystallinities, which is in turn related to water absorption and desorption in the polymer matrix. Therefore, one can assume that humidity affects the resistive switching characteristics of SPE-based atomic switches and ReRAMs. We investigated the effect of humidity on the resistive switching behavior of an Ag/Ag-PEO/Pt device [66]. Figure 5(a) plots the SET and RESET voltages as a function of the relative humidity (RH) level. Both of the operation voltages fell to almost half their magnitude as the RH level increased from ~30 to ~70%. The ON resistance maintained constant at the lower RH range, below 50%, but increased for higher RH levels. This increased ON resistance is attributed to the formation of a smaller filament, giving rise to the reduced structural stability of the filament. Therefore, the device showed both volatile and nonvolatile switching at 70% RH.

Figure 5(b) shows cyclic voltammetry (CV) curves measured in air (RH = 40–50%), for varied voltage sweep rates from 10 to 100 mV/s. The voltage sweep range was restricted to between 0.8 and −0.8 V, in order to investigate the electrochemical redox processes prior to the forming process. At 10 mV/s, a current peak was clearly observed at around −0.27 V during voltage sweep to the negative direction, which is assigned to the reduction from Ag^+^ to Ag [67]. As the sweep rate increased, this reduction peak became smaller and broader, and the device no longer exhibited any redox peaks at 100 mV/s, suggesting quasi-reversible or irreducible electron transfer electrode reactions at the Ag electrode. Figure 5(c) depicts CV curves obtained at different RH levels, ranging from 8% to 77%. At 8% RH, the device exhibited a small and broader reduction peak at around −0.15 V. As the RH level increased, this reduction peak became larger and sharper, with a slight shift to around −0.2 V. At 54% RH, another broad peak appeared at around 0.4 V, which can be attributed to oxidation from Ag to Ag^+^. As RH levels were increased further, both reduction and oxidation peaks were developed.

Under positive bias, an anodic (oxidation) reaction takes place at the Ag electrode, which is given by

(1) $$\mathrm{Ag}\to Ag^{+}+e^{-}.$$

Although counter charges (ClO_4_^−^ ions) already exist in the PEO matrix, counter electrode reactions are required at the Pt electrode to maintain charge electroneutrality to the anodic reaction (1). This is given by [41,68]

(2) $$H_{2}O+e^{-}\to OH^{-}+\frac{1}{2}H_{2}.$$

As a result, Ag^+^ and OH^−^ ions are generated in addition to the pre-existing Ag^+^ and ClO_4_^−^ ions. Figure 5(c) shows that the area of the hysteresis of the CV curve significantly increases at higher RH levels. The ion concentration *C*_*ion*_ generated by the anodic reaction can be calculated by the total generated charge in the positive voltage sweep and the volume of the PEO matrix. Figure 5(d) plots the calculated values of *C*_*ion*_ as a function of the RH level. The *C*_*ion*_ increases drastically at higher RH levels, which suggests that moisture absorption enhances both the anodic (1) and counter (2) reactions, resulting in the generation of a large number of Ag^+^ and OH^−^ ions. Such ionic charges separated at the two electrodes lead to the generation of an emf within the device, as illustrated in the inset of Figure 5(d). The inset of Figure 5(e) shows the equivalent circuit model of the device including the emf voltage *V*_*emf*_ with ionic resistance *R*_*i*_ and electronic resistance *R*_*el*_. Because of the high *R*_*el*_ of the PEO matrix, the cell voltage *V*_*cell*_, which is almost the same as *V*_*emf*_, is measured in actual measurements. The time evolution of *V*_*cell*_, measured after the anodic reaction, is plotted for various RH levels in Figure 5(e). The *V*_*cell*_ initially increased for the first 10 s and then decreased gradually over time, which is due to the equilibrium of ionic charges in the device. The maximum *V*_*cell*_ increased with increasing RH level, and became saturated at higher RH levels. This may be because the pre-existing Ag^+^ and ClO_4_^−^ ions partially cancel the emf voltage.

Figure 5(f) depicts Nyquist plots obtained from EIS measurements for a ~50-µm-thick Ag-PEO film sandwiched by Pt electrodes, at different RH levels. They are fitted with the equivalent circuit model, as illustrated in the inset, which is composed of a series resistance (R_s_), bulk response (R_b_/CPE_b_), and electrode polarization due to the formation of an electric double layer at the blocking Pt electrodes (R_ep_/CPE_ep_). The latter two components consist of a constant phase element (CPE) in parallel with a resistor (R). Figure 5(g) plots the electrical conductivity as a function of the RH level, which was calculated from the bulk resistance R_b_ (shown in the inset). The conductivity increases by more than one order of magnitude with increasing RH levels from ~30 to ~80%. At lower RH levels, the redox reactions at the Ag electrode are suppressed, due to the lowered counter reaction of the smaller amount of absorbed water molecules at the Pt electrode (Figure 5(h)). The suppressed oxidation rate and lower ionic conductivity result in larger SET and RESET voltages. As the RH level increases, the anodic reaction is enhanced with increased counter reactions of absorbed water molecules (Figure 5(i)). The conductivity of Ag ions oxidized at the Ag electrode and pre-existing Ag ions are also enhanced. As a result, the SET voltage is lowered at higher RH levels. The filament formed can be dissolved by a lower negative voltage, resulting in a lowered RESET voltage. This positively explains the operation voltages observed for varied RH levels, as shown in Figure 5(a). The proposed mechanism can be applied to resistive switching devices using other SPE materials.

## 5. Applications for SPE-based atomic switches

### 5.1. Flexible switch/memory devices

Owing to the mechanical flexibility of polymer materials, SPE-based atomic switches and ReRAMs were expected to be applied to flexible switch and memory devices at an early stage. Seung et al. fabricated Ag/PEO/PVK/Pt devices on a poly(ether sulfone) (PES) substrate [58]. By inserting a conductive PVK layer between PEO and Pt BE, the authors claimed that it becomes possible to control the length and diameter of Ag filaments in the PEO layer, which can in turn improve the switching characteristics. Hosseini et al. observed resistive switching in an Ag/Ag-doped chitosan/Pt device fabricated on a PES substrate under bending [59]. Kang et al. reported resistive switching of a Cu/Ti/PVP-PMF/Pt device on a polyethylene naphthalate (PEN) substrate, in which a thin Ti layer acts as a diffusion barrier for Cu diffusion [60]. The composition ratio of PMF to PVP controls the number of cross-linked chains, varying the number of sites for Cu-ion diffusion. They also demonstrated logic gate circuit operations using the two devices. Kim et al. observed stable switching of an Ag/Ag-doped CM:κ-car/Pt device fabricated on a poly(ethylene terephthalate) (PET) substrate under tensile and compressive bending [61]. Yang et al. demonstrated stable switching of an Ag/PEI/Pt device under repeated bending of a polyimide substrate, for a wide temperature range up to 150°C [62]. Recently, Zhang et al. reported stable resistive switching of an Ag/Ag salt-included PEI/Pt device under repeated hard bending of a PET substrate, maintaining a high ON/OFF resistance ratio [63]. In these studies, SPEs were spin-coated or drop casted onto plastic substrates.

Organic device fabrication processes utilizing spin-coating techniques are considered to be more cost effective and sustainable than processes that use more expensive lithographic techniques. However, this conventional method sometimes lacks the ability to restrict the deposition area and to control the thickness of coated films. If multiple devices share a spin-coated high-conducting electrolyte like SPE, the performance of each device is affected by its neighboring devices, which makes the evaluation of intrinsic characteristics difficult. To avoid such problems, we used a drop-on-demand inkjet printing method as an alternate method for the deposition of SPE solutions. Such inkjet printing can deposit the necessary amount of the target solution onto specific locations, thus providing an economical method of material delivery and a precise fabrication of micro-scale devices. This technique has been used for organic transistors [69,70], light-emission devices [71,72], and solar cells [73]. In most cases, organic materials with lower molecular weights were printed. On the other hand, for polymer materials with higher molecular weights, inkjet printing is a little difficult, and optimization of various parameters is necessary for the stable deposition of polymer solutions. In fact, there are few reports of the inkjet printing of high molecular weight polymer materials [74,75].

**Figure 6.**
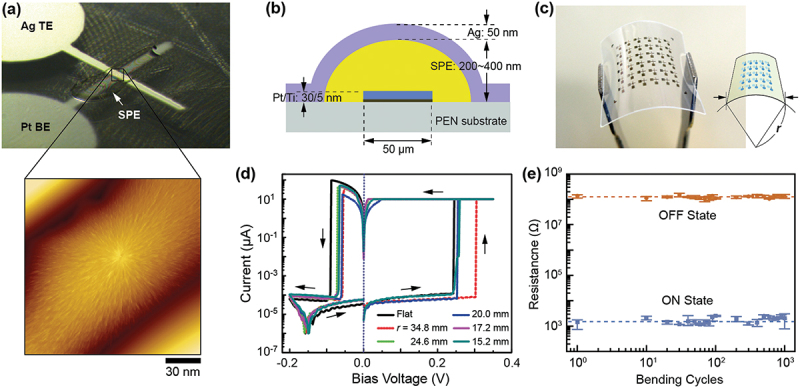
(a) Optical microscope image of a Ag/Ag-PEO/Pt device fabricated on a PEN substrate, and an AFM image of a Ag-PEO film inkjet-printed onto a Pt BE. (b) Schematic illustration of a cross-section of the fabricated device. (c) Photograph of the device and a schematic definition of the bending radius r. (d) I-V curves measured for different bending radii (e) Retention characteristics of the on and off resistance with respect to bending cycles. Reproduced with permission from [76], copyright 2021, American Institute of Physics [78], copyright 2015, Royal Society of Chemistry.

We successfully printed an SPE film in a reproducible way and fabricated Ag/Ag-PEO/Pt and Ag/PEO/Pt devices on a transparent, flexible PEN substrate [76–78]. After depositing Pt BEs, an Ag-PEO or PEO film was formed by dropping the SPE solution from an inkjet head. Many parameters were carefully optimized so as to realize stable deposition. These included the PEO molecular weight, SPE solution concentration, nozzle size, and the pulse width and height of the voltages applied to the inkjet head. The deposited solution initially spread out around the Pt BE, forming a coffee-ring pattern. Then, during evaporation of the solvent in air, the solution gradually coagulated on the Pt BE. Due to the difference in surface energies, the Pt BE behaves as hydrophilic patterns on the hydrophobic PEN surface. As a result, the SPE covered the Pt BE after drying. Atomic force microscopy (AFM) images of the deposited Ag-PEO film show a complex lamellar morphology, indicating its highly crystalline nature. Finally, the Ag TE was deposited so as to fabricate a cross-point structured device, as shown in Figure 6(a). The thickness of the Ag-PEO film was 200,400 nm, as illustrated in Figure 6(b).

The fabricated devices exhibited stable switching behavior under bending of the PEN substrate by applying pressure to both sides, where the bending radius was defined by a circular arc with a radius *r*, as illustrated in Figure 6(c). Figure 6(d) shows that the SET and RESET voltages were almost unchanged and without any deterioration under varied *r*, which is due to the high mechanical flexibility of the PEO matrix. The device also showed very stable ON and OFF resistance up to 10^3^ bending cycles when a high ON/OFF resistance rate was maintained at ~10^5^, as shown in Figure 6(e). These results indicate that SPE-printed atomic switches could be a promising candidate for flexible switch/memory applications.

### 5.2. Quantum conductance devices

When a conducting path is reduced to the atomic scale, the device exhibits QC, in which ballistic electron transport is dominant because the lateral dimension of the conducting path becomes comparable to the Fermi wavelength. In such circumstances, the devices generally present conductance steps at *nG*_*0*_ (*n* = 1, 2, 3, …), where *G*_*0*_ is the conductance of a single atomic point contact given by *G*_*0*_ *= 2e*^*2*^*/h* (*e* is the electron charge and *h* is Planck's constant). The miniaturization of semiconductor ICs in current ICT equipment has reached a stage where it is becoming technically difficult to realize these devices on the atomic scale [79]. On the other hand, due to their high scalability and low-power consumption, atomic switches can provide not only appropriate platforms for the investigation of fundamental quantum phenomena but also for the development of integrated quantum devices based on the nanoarchitectonics concept.

**Figure 7.**
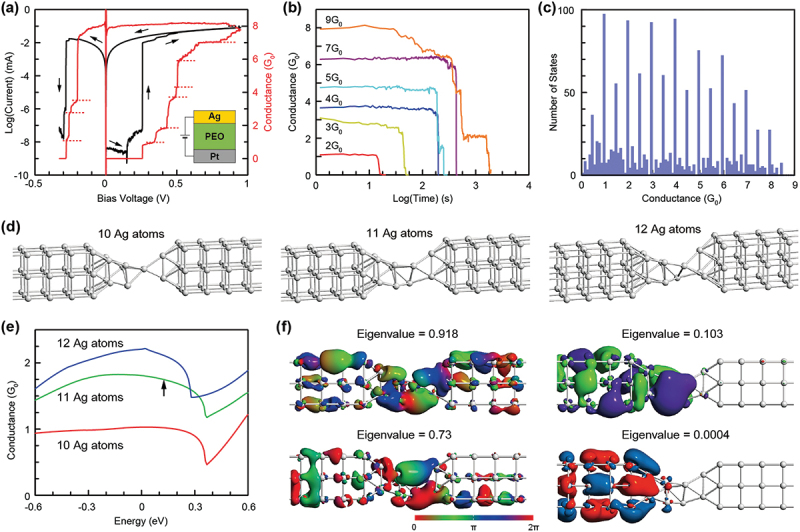
(a) *I-V* and the corresponding conductance curve of a Ag/PEO/Pt device, measured with *V*_*S*_ = 0.95 V. (b) Spontaneous conductance decay behavior, measured as a function of time, after realizing each conductance value. (c) Conductance-state histogram evaluated from the conductance curves collecting from various *V*_*S*_ of 0.2–0.8 V. (d) Geometrically optimized (relaxed) structures of different numbers (10, 11, and 12) of Ag atom chains connected between linked nine Ag atom blocks. (e) Energy-dependent conductance plot for different numbers of Ag atoms in the center region shown in (d). (f) Transmission eigenstates of 11 Ag atom chains forming a two-atom contact, calculated for the higher four eigenstates at 0.12 eV, as indicated by the arrow in (e). Reproduced with permission from [84] copyright 2017, Wiley.

QC was observed from the early stage of atomic switch research using Ag_2_S [7,80] and was subsequently observed in Cu_2_S-based atomic switches [81]. Oxide-based atomic switches also exhibited similar characteristics [82,83]. By carefully tuning the device structure and the experimental conditions, we succeeded in observing QC of SPE-based atomic switches at room temperature under atmospheric conditions [84,85]. Figure 7(a) shows a typical *I-V* (black) curve and a corresponding conductance-voltage (blue) curve for an Ag/PEO/Pt device with a 40 nm-thick PEO film. Under positive bias sweeping, the conductance curve increased in a stepwise fashion, as indicated by the dotted lines, and reached 8*G*_*0*_ at stop voltage *V*_*S*_ = 0.95 V. As the bias voltage was subsequently swept back, the device conductance was retained at the 8*G*_*0*_ level at 0 V and then decreased in a stepwise fashion in negative bias, showing nonvolatile switching behavior. When *V*_*S*_ was decreased, the device exhibited conductance states smaller than 2*G*_*0*_ and dropped before the bias voltage was swept back to 0 V, which corresponds to volatile switching behavior. This means that the observed QC state is closely related to the structural stability of the filament formed.

The stability of QC states was investigated by measuring the retention times of different conductance values, as shown in Figure 7(b). Conductance states of ≤ 1*G*_*0*_ dropped to zero immediately, and thus did not show any retention characteristics. In contrast, for conductance states of ≥ 2*G*_*0*_, the retention time became longer for higher conductance values and was likely to increase exponentially with the conductance values. The conductance-state histogram was determined by counting the conductance state as a plateau in the conductance-voltage curves for *V*_*S*_ = 0.2–0.8 V, which is summarized in Figure 7(c). The devices exhibit large peaks at integer multiples of *G*_*0*_, clearly demonstrating QC behavior. In addition, distinct peaks also appear at half-integer multiples of G_0_ with small fractional conductance variations. The state distribution range is determined by the maximum conductance of the largest *V*_*S*_ (~8*G*_*0*_ for *V*_*S*_ = 0.95 V). The device also exhibited an increase and decrease in the conductance in a stepwise fashion up to ~ 20*G*_*0*_ under positive and negative voltage pulse applications, respectively [86].

Controlling the QC is essential for designing and optimizing the desired characteristics of future atomic-scale devices. Detailed atomistic simulations were performed to understand the correlation between QC and the atomic point contact structure. QC originates from the narrowest region of a metal filament between electrodes, where the region consists of the number of atoms *n* (where, *n* = 1, 2, 3, …) participating in an atomic point contact. It was observed that the metal filament is composed of small Ag clusters that are precipitated in the PEO matrix [46]. Therefore, we considered an atomic point contact structure consisting of Ag atom chains between Ag blocks (of nine linked atoms) [84]. Under this configuration, the conductance states and their transmission eigenvalues and eigenstates of the entire structure were evaluated from first-principles density functional theory (DFT) simulations.

We calculated an atomic point contact where Ag atoms in the blocks are fixed on both sides but Ag atoms in the center region are relaxed to minimize the total energy. Figure 7(d) illustrates the geometrically optimized structures with three different numbers (10, 11, and 12) of Ag atoms in the center region. The results show that the 10-atom chain forms a single-atom point contact and exhibits almost 1*G*_*0*_ at E_F_, while the 11- and 12-atom chains form approximately two-atom point contacts with conductance values lower and higher than 2*G*_*0*_ at E_F_, respectively, as shown in Figure 7(e). The latter atomic contacts are heavily twisted and lose translational invariance along the channel. Therefore, electrons in the individual transmission channels suffer from heavy elastic scattering, and reduce the transmission rate (conductance) remarkably. The same two-atom point contacts can vary their conductance values, depending greatly on the atomic structure.

From the transmission channel analysis, it was revealed that the 11-atom chain possesses eight conducting channels at 0.12 eV above E_F_ (indicated by the arrow in Figure 7(e)). Of these channels, three with eigenvalues higher than 0.1 exhibit continuous wave functions between both blocks, whereas the remaining five, with eigenvalues lower than 0.001, do not reach the right block, as shown in Figure 7(f). In consequence, three partial transmission channels contribute to the total conductance. This indicates that the conductance of an atomic point contact is significantly influenced by the atomic configuration and a slight rearrangement of the atomic contact can easily cause half-integer multiples and fractional variations of the conductance state. The simulations also predicted that the atomic point contact is significantly altered by the presence of hydroxyl ions. If one or two hydroxyl ions are introduced near ideal single-atom chain and two-atom chain configurations, the atomic structures are immediately relaxed by incorporating hydroxyl ions and the corresponding conductance values are lowered. This suggests that moisture absorption in the polymer matrix affects the QC in the SPE-based atomic switch.

A similar QC was observed for an Ag/poly(3-hexylthiophene):[6,6]-phenyl-C61-butyric acid methyl ester/indium–tin oxide structure [57]. When the voltage sweep rate was decreased, conductance steps appeared at integer and half-integer multiples of *G*_*0*_. The former was ascribed to the formed Ag filaments with different atomic point contacts, while the latter was attributed to interactions between Ag filaments and hydrogen provided from the polymer matrix, which is consistent with our simulation results. A Cu/poly(1,3,5-trivinyl-1,3,5-trimethl cyclotrisiloxane (pV3D3)/Al device also showed both integer and half-integer QC under current sweeping up to 200 µA, which can be explained by the spin-split subbands of a Cu atomic contact [87]. Ag/Ag salt-included polyvinylimidazole (PVI)/Pt and Ag/Ag or Cu salt-included polyaniline (PANI)/Pt devices were found to exhibit QC behavior under voltage pulse applications [88,89]. Recently, Mallik et al. observed QC in a triptycene-based azo polymer (TBAP) film spin-coated on an Ag substrate using conductive-AFM (CAFM) with a Rh coated cantilever, which corresponds to a Rh/TBAP/Ag atomic switch [90].

### 5.3. Neuromorphic devices

**Figure 8.**
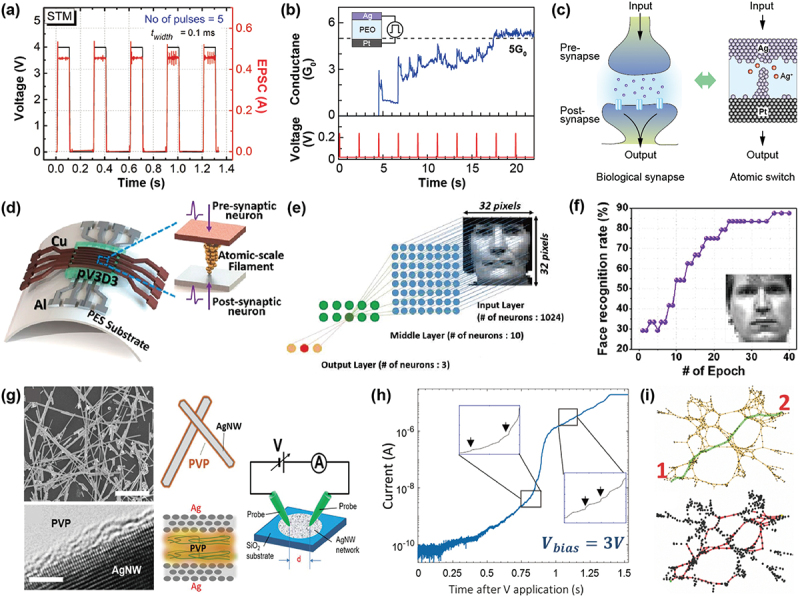
(a) STM behavior demonstrated by a Ag/Ag-PVI/Pt device. (b) Transition behavior from STM to LTM, demonstrated by a Ag/PEO/Pt device. (c) Schematic illustrating the resemblance between a biological synapse and an atomic switch. (d) Crossbar array consisting of Cu/pV3D3/Al devices, fabricated on a plastic substrate. (e) Two-layer perceptron ANN for face classification of 32 × 32 grayscale images. (f) Recognition rate as a function of the number of training epochs. (g) SEM image of PVP-coated Ag nanowire network and HR-TEM image showing a Ag nanowire coated with a nanometric PVP layer. Schematics illustrate a Ag/PVP/Ag atomic switch formed at an overlapped portion of nanowires, and the scheme of the measurement system. (h) Current vs. time measured for the nanowire network after applying a constant voltage between probes. Two insets showing an enlarged section of the activation features during two different time regions. (i) Upper: graph representation of an electrical network simulated. The black dots represent opened junctions. The edge drawn as a green line represent the shortest topological path between probes labeled ‘1’ and ‘2’. Lower: distribution of activated junctions just before the network reaches a high-conductance state. The red lines represent the edges of low-resistance channels. Reproduced with permission from [84] copyright 2017, Wiley [86], copyright 2022, Wiley [87], copyright 2019, American Chemical Society [94], copyright 2019, Nature.

As mentioned in the Introduction, there has been increasing interest in the creation of non-software-specific artificial intelligence (AI) functions, utilizing hardware that takes advantages of material characteristics. Various devices and systems have been proposed to mimic the behavior of synapses and neurons using RRAMs, although it is beyond the scope of this review to describe them all. For detailed information, readers can refer to a number of excellent review papers [29,91–93]. As for SPE-based atomic switches, Krishnan et al. demonstrated synapse-like memory characteristics in an Ag/Ag-PVI/Pt device under voltage pulse applications, which are similar to the short-term and long-term plasticity of biological synapses, as shown in Figure 8(a) [88]. Using such memory functions, they also demonstrated that 5 × 5 Ag/Ag-PVI/Pt arrays can mimic the learning/forgetting behavior for input images depending on the voltage pulse conditions. Higuchi et al. investigated short-term memory (STM) and long-term memory (LTM) behaviors of Ag/Ag (or Cu)-PANI/Pt devices in which the pulse voltage conditions were tuned [89]. CAFM-based Ph/TBAP/Ag atomic switches exhibited a gradual transition from STM to LTM under repeated voltage sweeping at varied interval times [90].

We also observed a similar STM to LTM transition behavior in an Ag/PEO/Pt atomic switch under consecutive voltage pulse applications, as shown in Figure 8(b) [86]. The device initially did not show a response to the input stimulus (which corresponds to sensory memory [95]), but increased its conductance after the third pulse (corresponding to STM), and finally maintained a certain QC value (corresponding to LTM). After the input pulse was stopped, said conductance state could remain for a certain time, ranging from 30 s to several minutes, depending on the QC value. The structural stability of an Ag filament is strongly dependent on the repetition rate and intensity of the stimulating pulses, which mimics the signal transmission of biological synapses, as illustrated in Figure 8(c). An important point to bear in mind here is that such synaptic behavior is emulated by a single atomic switch. For CMOS devices to achieve the same behavior would require them to have more than 10 transistors, which would result in larger devices and increased power consumption. Thus, the atomic switch has great potential for use as an essential building block in neural computing systems.

Atomic switches can be used for artificial neural networks (ANNs) that are usually composed of a crossbar array, in which the respective devices are connected in a grid pattern, with each row and column representing a different synapse or neuron. Jang et al. simulated face recognition capabilities of an ANN using Cu/pV3D3/Al devices, as shown in Figure 8(d) [87]. The ANN was based on a two-layer perceptron model, which is designed with a crossbar architecture with 32 × 32 input neurons, 10 middle neurons, and 3 output neurons, as illustrated in Figure 8(e). After training with nine 32 × 32 pixel images of three people, extracted from the Yale Face Dataset, the rate of the face recognition was calculated by applying nine images of three people to the input of the ANN. Figure 8(f) shows that the recognition rate reaches 88% after 36 epochs, which is comparable to the results obtained for a one-layer perceptron model with an array consisting of TiN/TaO_x_/HfAlO_x_/TiN RRAM devices [96]. The ANN architecture with atomic switches allows for fast and efficient processing of data and has the potential to be used as neuromorphic computing systems with high density and scalability.

Neuromorphic functions can be achieved by atomic switches embedded in complex self-assembled nanostructures. Diaz-Alvarez et al. reported the signal transmission behavior of a randomly self-assembled Ag nanowire network, in which each nanowire is coated with a nanometric PVP layer, as shown in Figure 8(g) [94]. The network was formed by drop casting a nanowire solution onto a SiO_2_ substrate. Figure 8(h) presents a typical *I-t* response acquired on the network connected by two fixed probes under a constant voltage bias larger than a threshold, showing activation features in the transition from a low-conductance state to a high-conductance state. The activation sequence, as indicated by arrows in the insets, corresponds to spatially and temporally random connections and disconnections within the network, resulting from the formation and dissolution of Ag filaments in Ag/PVP/Ag atomic switches formed at overlapped portions of nanowires. Figure 8(i) consists of graph representations of the Laplacian matrix of an electrical network, in which black dots and red lines represent opened junctions (atomic switches in OFF states) and edges of activated junctions (atomic switches in ON states), respectively. In the initial state, with all junctions opened, the inhomogeneous density of the nanowire network produces an irregular voltage distribution. Junctions in denser areas switch in an avalanche-like process, bridging a low resistance pathway, and such a single pathway of activated junctions forms a connected channel. Finally, low resistance channels are formed in parallel, as seen in the lower part of Figure 8(i), and the network reaches the high-conductance state. Using this characteristic, the nanowire network exhibits complex dynamics arising from multi-scale interactions of atomic switches. These include a corrective memory response, different power-law fluctuation scalings, and reconfiguration behavior, all of which are similar to the synaptic plasticity of the brain. This type of atomic switch network has great potential for use in the hardware implementation of natural computing [97].

## 6. Summary and outlook

**Figure 9.**
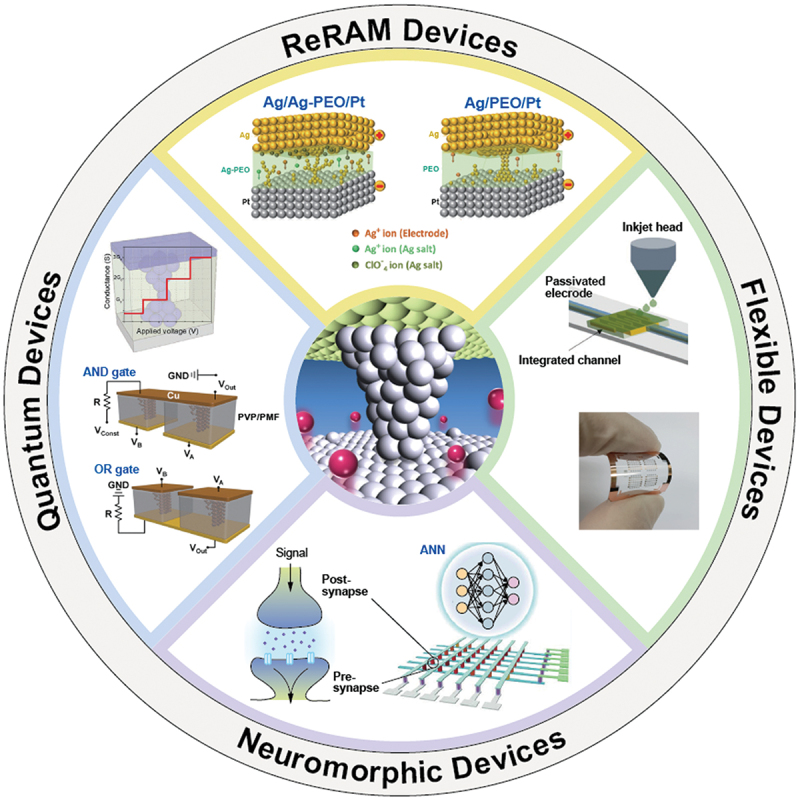
Potential applications of SPE-based atomic switches. In addition to bi-stable resistive switching memories, SPE-based atomic switches have excellent potential for use in flexible devices, including printed transistors and sensors, quantum devices including multilevel memories and logic-gate circuits, and neuromorphic devices such as artificial synapses and ANN circuits. Reproduced with permission from [39], copyright 2016, Wiley [60], copyright 2017, American Chemical Society [98], copyright 2022, Wiley.

This review focuses on describing the resistive switching mechanism of Ag salt-included SPE-based atomic switches and their device applications. The potential applications for SPE-based atomic switches (and ReRAMs) covers a wide range, from ICTs to AI, as summarized in Figure 9. The observed resistive switching is attributed to the formation and dissolution of an Ag filament between the active and inert electrodes, based on Ag-ion transport in the polymer matrix and redox reactions at the electrodes. The devices exhibit repeatable switching cycles with high ON/OFF ratios and low power consumption, and show volatile and nonvolatile switchings depending on the measurement and environmental conditions. The high conductivity of Ag ions enabled us to observe the fundamental filament growth processes in planar devices, even with a micrometer gap, and to elucidate the kinetic factors to determine switching characteristics such as the redox reaction rate at the electrode interfaces, ionic mobility in the polymer matrix, and reduction sites for precipitations. It was also found that the resistive switching behavior depends strongly on the crystallinity of the polymer matrix materials employed. Moisture absorption in the polymer matrix (PEO) also plays a crucial role in determining the device performance. In other words, controlling residual water in the polymer matrix is important in obtaining the desired characteristics.

Compared to other electrolyte materials, such as metal oxides and chalcogenides, SPEs have several advantages for atomic switches, which we describe in this review. The electrically insulating and high ionic conductivity of SPEs provides an electrochemically ideal switching matrix, in which switching behavior is caused by the smooth migration of metal ions and electrochemical reactions in a controlled way. In contrast, the formation of metal filaments in the oxide matrix is stochastic and, because of the low ionic conductivity, it is somewhat difficult to control the resistive switching behavior. To improve controllability, the thickness of the metal oxides should be reduced to 20 nm or less, and it is sometimes necessary to tune the film structure [67]. On the other hand, chalcogenides such as Ag_2_S and Cu_2_S are difficult to apply to the Si CMOS fabrication processes, because sulfur becomes an impurity in semiconductor ICs. Their relatively high electronic conductivity may also be a disadvantage in terms of power consumption of the OFF states. However, SPEs also have the disadvantage of being difficult to apply with lithography processes, because the matrix polymer is easily dissolved or damaged. There have recently been reports of attempts to directly pattern spin-coated PVA films using electron-beam lithography [99], but this method needs to be investigated to determine whether it can be applied to SPE-based atomic switches.

Regarding potential SPE-based atomic switch applications, we first showed a flexible atomic switch fabricated on a plastic substrate, in which the Ag-PEO electrolyte was inkjet-printed. By optimizing various parameters and utilizing the difference in surface energy between the substrate and BE, reproducible printing of SPE onto targeted electrodes was achieved. The fabricated devices exhibited stable resistive switching behavior with a maintained high ON/OFF resistance ratio, even under repeated light bending of the substrate. Inkjet printing is one of the most important fabrication techniques in the field of printed electronics [100]. Inkjet printing of SPE solutions is still challenging, but offers great potential for fabricating micro-scale devices without using lithography processes, which may modify the properties of the polymer matrix. In addition, if electrodes could be formed using metallic nanoparticle inks instead of by vacuum deposition, it would be possible to realize low-cost, fully printed atomic switches and their circuits in open air.

We also demonstrated QC in an atomic switch with a thin PEO film. By carefully tuning voltage stimuli conditions, reproducible QC was observed at room temperature in the atmosphere. First-principles DFT simulations revealed which atomic configurations are geometrically optimized and the stability of such configurations for various conductance states, interpreted by the transmission channels for atomic point contacts. The simulations also predicted that hydroxyl ions could stabilize atomic point contact structures, again implying the importance of moisture absorption in the polymer matrix. The observed QC can provide the basis for multilevel data storage in a single memory device. Because device operation relies on the formation of a single filament, ultrahigh-density memory integration is possible in a three-dimensional stacked structure with CMOS back-end processes [98]. The QC can be used for logic gates and circuits, as demonstrated by Ag_2_S-based atomic switches [7] and PVP/PMF-based ReRAMs [60], which in turn would become building blocks for in-memory computing architectures.

Finally, SPE-based atomic switches also exhibit neuromorphic behaviors that mimic biological synapses and neurons, not only on a single device level but also on a large-scale level, by utilizing the structural stability of the metal filaments formed. A single switch shows short-term and long-term synaptic plasticity, depending on the bias voltage conditions. Such atomic switches can be used in a crossbar array for the implementation of ANNs. In self-assembled switch networks, more complex dynamical signal processing is observed by current path formation or disruption due to the randomly occurring SET/RESET events of individual switches, which mimic the neural dynamics of the human brain. To develop intelligent nanoarchitectonic systems using SPE-based atomic switches, further investigations are required to obtain a reliable, low-power consumption, reproducible readout of intermediate conductance states, and a detailed understanding of dynamical behavior in large-scale switch networks.
